# Identification of differentially expressed microRNAs in human male breast cancer

**DOI:** 10.1186/1471-2407-10-109

**Published:** 2010-03-23

**Authors:** Ulrich Lehmann, Thomas Streichert, Benjamin Otto, Cord Albat, Britta Hasemeier, Henriette Christgen, Elisa Schipper, Ursula Hille, Hans H Kreipe, Florian Länger

**Affiliations:** 1 Institute of Pathology, Medizinische Hochschule Hannover, D-30625 Hannover, Germany; 2 Department of Clinical Chemistry/Central Laboratories, Universitätsklinikum Hamburg-Eppendorf, D-20246 Hamburg, Germany; 3 Department of Gynecology, Medizinische Hochschule Hannover, D-30625 Hannover, Germany

## Abstract

**Background:**

The discovery of small non-coding RNAs and the subsequent analysis of microRNA expression patterns in human cancer specimens have provided completely new insights into cancer biology. Genetic and epigenetic data indicate oncogenic or tumor suppressor function of these pleiotropic regulators. Therefore, many studies analyzed the expression and function of microRNA in human breast cancer, the most frequent malignancy in females. However, nothing is known so far about microRNA expression in male breast cancer, accounting for approximately 1% of all breast cancer cases.

**Methods:**

The expression of 319 microRNAs was analyzed in 9 primary human male breast tumors and in epithelial cells from 15 male gynecomastia specimens using fluorescence-labeled bead technology. For identification of differentially expressed microRNAs data were analyzed by cluster analysis and selected statistical methods.

Expression levels were validated for the most up- or down-regulated microRNAs in this training cohort using real-time PCR methodology as well as in an independent test cohort comprising 12 cases of human male breast cancer.

**Results:**

Unsupervised cluster analysis separated very well male breast cancer samples and control specimens according to their microRNA expression pattern indicating cancer-specific alterations of microRNA expression in human male breast cancer. miR-21, miR519d, miR-183, miR-197, and miR-493-5p were identified as most prominently up-regulated, miR-145 and miR-497 as most prominently down-regulated in male breast cancer.

**Conclusions:**

Male breast cancer displays several differentially expressed microRNAs. Not all of them are shared with breast cancer biopsies from female patients indicating male breast cancer specific alterations of microRNA expression.

## Background

Breast cancer in men accounts for approx. 1% of all breast cancers [1]. Despite a statistically significant raise in the incidence of male breast cancer over the last 25 years it is still a quite rare disease. Therefore, therapy is mainly based on what is known from female breast cancer. Despite the fact that randomized controlled prospective trials are not possible due to the low incidence, it is clear from retrospective analyses that male breast cancer is not exactly the same entity as female breast cancer [2].

The discovery of a new class of small non-protein-coding RNAs with pleiotropic regulatory functions ("microRNAs") has substantially changed our understanding of tumor development and progression [3]. New molecular mechanisms, new diagnostic markers and new potential therapeutic targets have been discovered during the last years in the field of microRNA research. Consequentially, several studies revealed specific changes in microRNA expression in female breast cancer, the most frequent malignancy in females (see [4] and references therein). But so far only a single study, conducted nearly in parallel to the work described inhere, analyzed the expression of microRNA genes in human male breast cancer [5].

Therefore, we analyzed the expression pattern of 319 microRNAs in 9 male breast cancer specimens as wells as in 15 epithelium fractions from male gynecomastia specimens using fluorescence-labeled bead technology from Luminex™. Results were validated for the most differentially regulated microRNAs in these 9 tumors, the control samples and an independent test series of 12 male breast cancer specimens using stem-loop primer based real-time PCR methodology (TaqMan™ assays from Applied Biosystems).

Only formalin-fixed paraffin-embedded (FFPE) specimens were available for this study. However, in a previous study we could demonstrate that RNA extracted from FFPE specimens is a suitable substrate for microRNA profiling using fluorescence-labeled bead technology from Luminex™ [6].

## Results

### Characterization of the study cohort

For the comprehensive determination of the microRNA expression profile of human male breast cancer 9 specimens were retrieved from the archives. The primary selection criteria were tumor size and tumor cell content, because several microgram of total RNA are required for the full profiling with five different bead pools (see Materials and Methods). All tumors were grade 2 or 3, with a mean proliferation rate of 20% (as determined by MIB1 labeling, median: 20%, range: 10 - 40%). They were all estrogen and androgen receptor positive, all but one were also progesterone receptor positive, but negative for Her2 overexpression, p63 expression and expression of basal cytokeratines 5 and 14, thus representing typical male breast cancer cases [1].

The 12 additional cases for independent validation were comprised by 6 primary invasive carcinomas, 3 local recurrences, and 3 metastases. The distribution of tumor grade, proliferation rate and the immunohistochemically determined tumor marker profiles were very similar to the first nine cases under study. A detailed description of all 21 specimens is provided in Table 1.

**Table 1:** Overview of all cases analysed in this study

	age		grade	ER	PR	AR	MIB	Her2	p63	CK5/14
**MBC1**	61	DIC	2	8	3	7	30	1	-	-
**MBC3**	63	DIC	3	6	6	5	25	0	-	-
**MBC6**	64	DIC, rec	2	5	0	7	40	2	-	-
**MBC8**	62	DIC	2	6	8	5	10	1	-	-
**MBC9**	53	DIC	3	7	8	8	25	1	-	-
**MBC10**	47	DIC	2	8	5	7	20	1	-	-
**MBC11**	51	DIC	2	5	5	5	15	0	-	-
**MBC12**	74	DIC	2	7	8	6	10	0	-	-
**MBC13**	71	DIC	2					0		
MBC2	62	Met		8	6	6	10	0	-	-
MBC4	69	Met		4	6	5	30	1	-	-
MBC5	66	DIC, rec	2	6	0	4	20	0	-	-
MBC7	53	Met		8	7	7	15	0	-	-
MBC15	68	DIC, rec	2	8	8	6	15	0	-	-
MBC16	63	DIC	3	8	7	6	20	2	-	-
MBC20	70	DIC	2	6	4	6	10	2	-	-
MBC22	41	DIC	3	7	7	6	15	0	-	-
MBC23	69	DIC	3	6	4	4	20	1	-	-
MBC24	67	DIC	3	5	7	5	35	0	-	-
MBC26	55	DIC	3	7	8	6	10	0	-	-
MBC27	83	inv.pap.	2	7	4	7	40	3	-	-

bold: used for the comprehensive profiling

ER, PR, and AR staining was scored according to the Allred score

MBC5 is a local recurrence of the primary carcinoma MBC3

MBC13: specimen was no longer available for further characterization, therefore incomplete marker profile

For comparison a series with 15 male gynecomastia specimens was analyzed representing a benign proliferative condition of the ductal epithelium.

### microRNA profiling of human male breast cancer

The expression of 319 microRNAs was analyzed in 9 primary human male breast cancer specimens using fluorescence-labeled bead technology. As a control the expression of these 319 microRNAs was measured in 15 epithelial cell fractions from male gynecomastia specimens, which were pooled in 4 mixes due to low RNA yield.

**Figure 1:**
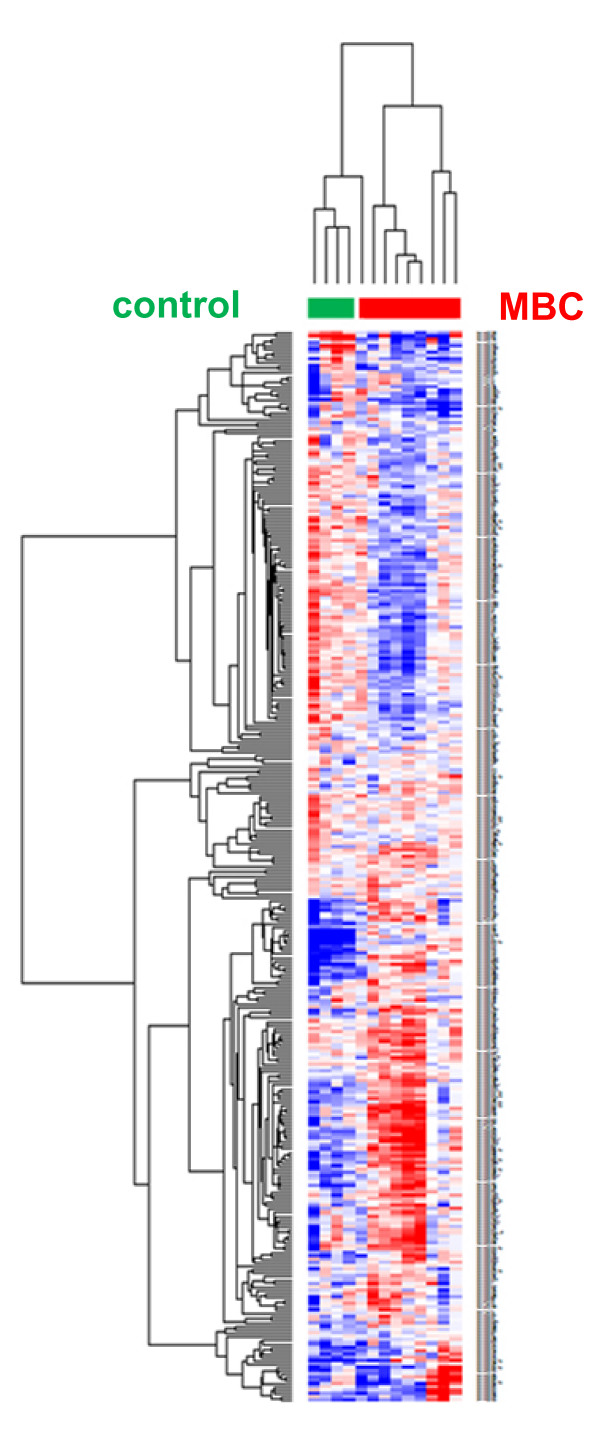
**Unsupervised cluster analysis of the expression level of 319 microRNAs in 9 male breast cancer and 4 pools of gynecomastia specimens (pools of 3 or 4 individual samples each)**. For this analysis the normalization was performed as described by Blenkiron et al. [26] with inter-pool normalization.

Unsupervised clustering of all microRNA expression data separated very well the carcinoma specimens from the pools of control samples, clearly indicating cancer-specific alterations of microRNA expression in male breast cancer (Figure 1).

**Figure 2:**
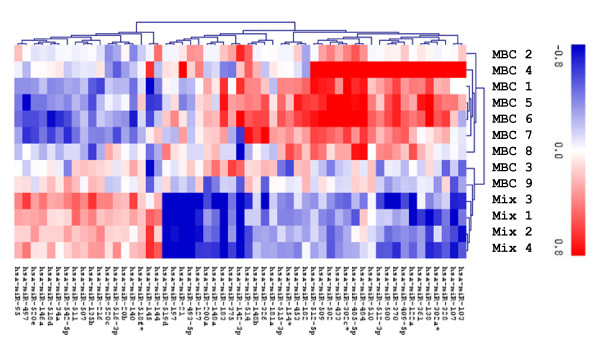
**t-test (p = 0.01) for the identification of differentially expressed microRNAs in human male breast cancer**.

T-test based analysis for comparison of carcinoma and normal samples identified several differentially expressed microRNAs (Figure 2), among them miR-21 reported to be overexpressed in female breast cancer and miR-145 reported to be down-regulated in female breast cancer [7,8]. Up-regulation of miR-519d, miR-183, miR197, and miR-493-5p (more than twofold in comparison to gynecomastia, see Table 2) has not been reported so far in female breast cancer.

**Table 2:** List of microRNAs differentially expressed in human male breast cancer

UID	Mean MBC (log2)	Mean Mix (log2)	FoldChange	p-value
**hsa-miR-21**	-0,03	-2,38	5,11	0,004029143
**hsa-miR-519d**	0,05	-1,46	2,85	0,004768175
**hsa-miR-183**	0,23	-1,26	2,82	0,003398121
**hsa-miR-197**	0,01	-1,03	2,05	0,002805108
**hsa-miR-493-5p**	-0,07	-1,08	2,02	0,003438789
**hsa-miR-142-3p**	0,00	-0,98	1,98	0,00169614
**hsa-miR-138**	0,33	-0,62	1,93	0,00024239
**hsa-miR-127**	0,12	-0,78	1,87	0,00040025
**hsa-miR-370**	0,28	-0,60	1,84	0,005644958
**hsa-miR-122a**	0,19	-0,66	1,81	0,00143915
**hsa-miR-514**	0,36	-0,49	1,80	0,000596294
hsa-miR-302c*	0,48	-0,34	1,76	0,001330654
**hsa-miR-502**	0,35	-0,45	1,74	0,000582627
**hsa-miR-107**	-0,01	-0,77	1,69	0,000812296
**hsa-miR-485-5p**	0,48	-0,25	1,66	0,001501971
**hsa-miR-500**	0,22	-0,50	1,64	0,00600779
**hsa-miR-433**	0,32	-0,36	1,60	0,000983356
**hsa-miR-148a**	0,05	-0,62	1,59	0,000878915
**hsa-miR-509**	0,37	-0,24	1,53	0,000637106
**hsa-miR-409-5p**	0,19	-0,38	1,48	0,000646352
**hsa-miR-512-5p**	0,33	-0,22	1,46	0,002213783
**hsa-miR-453**	0,28	-0,26	1,45	0,000888411
**hsa-miR-200a**	0,05	-0,48	1,44	0,000906633
**hsa-miR-328**	0,13	-0,38	1,42	0,003384969
**hsa-miR-103**	-0,08	-0,60	1,42	0,000858599
**hsa-miR-375**	0,06	-0,44	1,41	0,002278685
hsa-miR-154*	0,14	-0,35	1,40	0,002861893
**hsa-miR-182**	0,24	-0,23	1,38	0,004889343
**hsa-miR-515-3p**	0,16	-0,30	1,38	0,00254786
**hsa-miR-148b**	0,27	-0,19	1,37	0,000346533
**hsa-miR-365**	0,00	-0,31	1,23	0,003164365
**hsa-miR-181a**	0,05	-0,21	1,20	0,006523063
**hsa-miR-510**	0,21	-0,04	1,19	0,005192826
hsa-miR-518f*	-0,03	0,20	-1,17	0,001226005
**hsa-miR-20b**	-0,12	0,13	-1,19	0,004383712
**hsa-miR-520h**	-0,15	0,13	-1,22	0,009803676
**hsa-miR-412**	-0,18	0,14	-1,24	0,005788243
**hsa-miR-34a**	-0,21	0,14	-1,27	0,005886412
**hsa-miR-520c**	-0,14	0,22	-1,28	0,004330261
**hsa-miR-507**	-0,12	0,27	-1,31	0,00018419
**hsa-miR-518d**	-0,22	0,20	-1,34	0,004924138
**hsa-miR-95**	-0,16	0,27	-1,35	0,000709481
**hsa-miR-146a**	-0,19	0,25	-1,35	0,004129367
**hsa-miR-516-3p**	-0,23	0,23	-1,38	0,000300035
**hsa-miR-216**	-0,14	0,32	-1,38	0,000500204
**hsa-miR-544**	-0,23	0,24	-1,38	0,009547717
**hsa-miR-511**	-0,13	0,34	-1,39	0,003257481
**hsa-miR-542-5p**	-0,25	0,24	-1,40	0,001777145
**hsa-miR-144**	-0,16	0,33	-1,41	0,004720278
**hsa-miR-520e**	-0,25	0,25	-1,41	0,000266494
**hsa-miR-140**	-0,28	0,28	-1,47	0,00867657
**hsa-miR-135b**	-0,25	0,40	-1,57	0,000641931
**hsa-miR-497**	-0,29	0,36	-1,57	0,001634003
**hsa-miR-145**	-0,37	0,44	-1,75	0,007261167

A detailed analysis revealed 33 statistically significantly up-regulated and 21 statistically significantly down-regulated microRNAs in human male breast cancer in comparison to male gynecomastia specimens (Table 2). Strongest up-regulation was found for miR-21, already described to be significantly up-regulated in female breast cancer ([4] and references therein) and other solid tumors. ([3] and references therein)

### Validation of differentially expressed microRNAs using real-time PCR

In order to validate the results of the profiling experiments, the expression level of the two most up-regulated (miR-21, miR-519d) and the two most down-regulated (miR-497, miR-145) microRNAs were validated in the carcinoma training cohort (n = 9), an independent carcinoma test cohort (n = 12) and the four control samples using real-time PCR methodology (stem-loop primer-based kits from Applied Biosystem). Correlation analysis revealed a good correlation between the real-time PCR data (stem loop primer PCR) and the Luminex™ data (fluorescence labeled beads) for miR-21 (r = 0.93), miR-145 (r = 0.513), and miR-497 (0.71).

**Figure 3:**
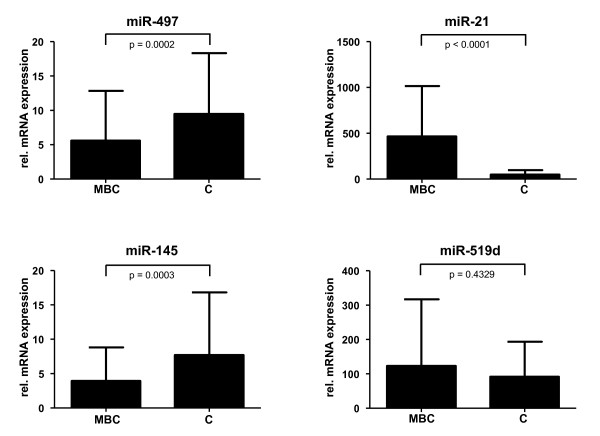
**Expression of the two most strongly up-regulated and the two most strongly down-regulated microRNAs in male breast cancer and control samples as determined by real-time PCR**. Relative expression levels for every sample were calculated by normalizing to the mean expression level of the three small RNA species RNU24, U54, and Z-30. All measurements were performed independently two times. Statistical significance of differences between cancer (MBC) and control samples (C) were calculated using Mann-Whitney-U-test.

Welch-based t-test demonstrates a statistically significant difference between carcinoma samples and the controls for miR-21, miR-145, and miR-497, but only a trend for miR-519d (Figure 3).

### Donwregulation of miR-21 target genes in male breast cancer

**Figure 4:**
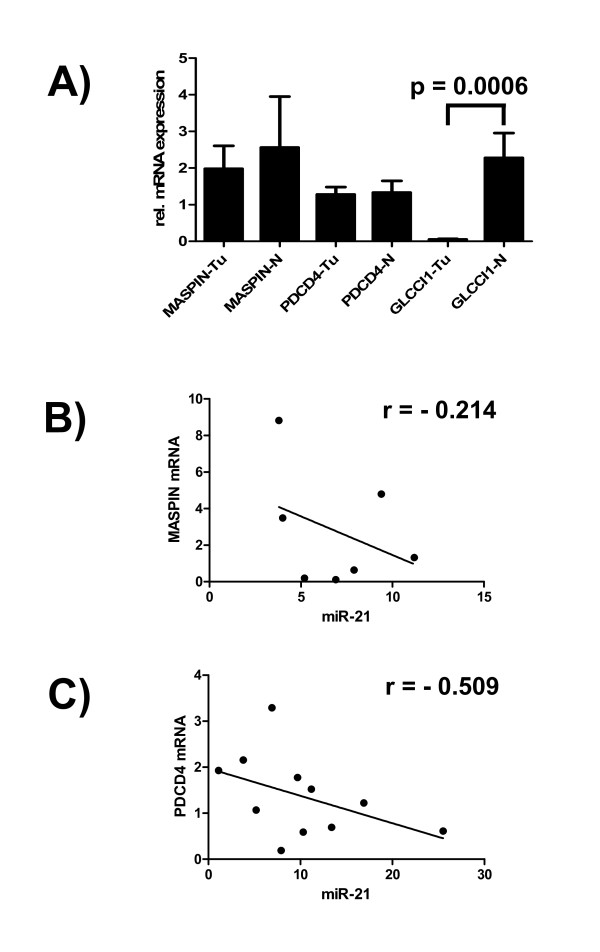
**Expression of miR-21- target genes in male breast cancer in comparison to the control samples (A), correlation of miR-21 expression with the mRNA level of MASPIN (B) and PDCD4 (C) in male breast cancer specimens**. Relative expression levels for every sample were calculated by normalizing to the mean expression level of two reference genes (*βGUS *and *TBP*). For every target gene the mean of the expression level in the control group was set equal to 1. Every individual measurement in the tumor samples was then normalized to this expression level. All measurements were performed independently two times. Error bars indicate standard deviation of the mean.

To further explore our findings, we analyzed the expression of several target genes of miR-21. We concentrated on miR-21, because it is the most significantly up-regulated miR-21 in our study and several validated target genes are described in the literature (see [9] and references therein, see also the new database miRecords [10]). Quantitative real-time RT-PCR was employed to measure mRNA levels of target genes, because quantification of protein expression levels in archival clinical specimens is difficult and depends on the availability of suitable antibodies. Mean expression in carcinoma specimens compared to the control group was measured for MASPIN, PDCD4, GLCCl1, BMPR-II, and APAF-1. Whereas no clear differences were discernible for BMPR-II and APAF-1, respectively (data not shown), differential expression, albeit to a different extent, was found for MASPIN, PDCD4 and GLCCl1 (Figure 4). GLCCl1 showed a highly significant reduction in the tumor specimens in comparison to the control group. MASPIN showed only a weak reduction whereas PDCD4 didn't show any difference in this presentation of the data (Figure 4A). However, if the mRNA expression level is correlated to the miR-21 expression level case-by case, a clear inverse correlation is discernible for MASPIN (Figure 4B), and much more pronounced for PDCD4 (Figure 4C), one of the best studied target genes of miR-21 [9,11]. Nearly all measurements for PDCD4 are within the 95% confidence interval (data not shown).

## Discussion

Cluster analysis of microRNA expression profiles revealed cancer-specific alterations of microRNA expression in human male breast cancer. Many, but not all of these alterations are shared with female breast cancer of the ductal invasive type. From the 20 most differentially regulated microRNAs (10 most up-regulated and 10 most down-regulated, see Table 2) 7 are shared with female breast cancer (miR-21, miR-127, miR-122a, miR-135b, miR-140, miR-497, miR-145). All 10 most up-regulated microRNAs are already reported to be expressed at an elevated level in human malignancies [7,12-17]. The most strongly up-regulated microRNA miR-21 is described by several studies to be markedly up-regulated in various epithelial malignancies, among them female breast cancer [18]. The most prominently down-regulated microRNAs miR-145 and miR-497 are already identified as down-regulated in female breast cancer [7,8]. Further studies attribute already quite well characterized tumor-suppressor function to miR-145 and miR-497 in other malignancies as well [16,19,20]. miR-145, whose most prominent validated target gene is c-myc [21], was already found to be down-regulated in male breast cancer by Fassan et al. [5].

Employing quantitative real-time RT-PCR a clearly discernible negative correlation between miR-21 and miR-21 target gene expression could be demonstrated (Figure 4). However, not all reported miR-21 target genes tested in this study showed reduced mRNA expression in male breast cancer specimens indicating tumor type specific differences and a more complex regulation under many circumstances.

A recent publication describes the kallikrein family of serine proteases as potential targets of miR-519d, the second most prominently up-regulated microRNA in male breast cancer [22]. However, the identification of target genes specific for miR-519d is especially challenging because it shares target sequence specificity with miR-17-5p, miR-20 A and B, as well as miR-106 A and B. Other microRNAs overexpressed more than twofold in human male breast cancer, but not yet described in female breast cancer are miR-183, miR-197, miR-493-5p, and 519d. However, these microRNAs are described as up-regulated in other human malignancies [12,14-16]. miR-183 is destabilizing a ubiquitine-ligase (βTrCP1, ref. [23]), miR-197 targets the tumor-suppressor FUS1 [15], whereas for miR-493-5p convincing experimental data for a validated target gene are still missing. The most prominent target gene with a high score predicted by TargetScanHuman (release 5.1, http://www.targetscan.org) is FOXP2 which plays an important role in neural mechanism involved in language development [24].

A limitation of this study might be that completely normal male breast epithelium from healthy individuals is principally not available in sufficient amounts for molecular studies. Therefore, we chose male gynecomastia specimens for comparison which represent a condition of increased but benign ductal epithelial proliferation [25]. This serves as a suitable reference for the identification of alterations in microRNA expression due to malignant hyperproliferation, which takes place in male breast cancer. This approach has the advantage that it filters out all simply proliferation associated changes in microRNA expression.

A conceptual advantage of the Luminex™ approach is the omission of any amplification step, which might introduce a bias of unknown extent which is difficult to control. The hybridization of probe and target sequence in homogenous solution circumvents also all potential artifacts due to the inhomogeneous reaction conditions at the boundary surface of a microarray. A potential disadvantage is the fact that the hybridization takes place in five separate tubes. This requires inter-pool normalization which might introduce a bias as well (see below). It has also to be mentioned that in comparison to PCR-based approaches quite a lot of total RNA is required.

It was decided to perform inter-pool normalization as described by Blenkiron et al. [26], because the spike-in controls provided by Luminex™ and contained within every pool showed some variation between the five pools. A detailed analysis for selected targets comparing this normalization strategy with the one recommended by Luminex™ (based on the spike-in controls) showed that for individual microRNAs expression patterns depend on the normalization strategy. But the expression patterns of the most differentially regulated genes (validated independently by real-time PCR, see Figure 3) were independent from the normalization strategy (data not shown).

During the preparation of this manuscript Fassan et al. published the microRNA expression profiling of 23 male breast cancer specimens using a published microarray platform [5]. Since the analytical platforms (microarray versus fluorescence labeled beads with a different selection of represented microRNAs) and the study design are different (all samples analyzed with the microarray in Fassan etal. versus independent validation of results obtained with the training set in a test set), the results are not directly comparable. Therefore, these studies lead to somewhat different results, though very similar in scope and size.

The most prominently up-regulated microRNA in our study is the well-known miR-21, described by several studies to be up-regulated in female breast cancer as well as in other solid tumors ([3] and references therein). This finding serves as a very good internal control for our findings since the overexpression of this microRNA in male breast cancer is an expected finding. Interestingly, miR-21 was not found to be differentially expressed in male breast cancer by Fassan et al.

A difference between the breast cancer series used by Fassan et al., which is described in detail in a previous publication [27], and the training and test cohort used in this study is the prevalence of basal cytokeratine (CK5/6 and CK14) expression. The total absence of basal cytokeratines in our series is contrasted by nearly 20% of cases positive for basal cytokeratines in the series used by Fassan etal. This might explain differences in microRNA expression patterns. Therefore, in the future a multi-institutional large-scale profiling approach employing different methodologies for the analysis of the very same set of samples is desirable.

## Conclusion

In summary, we describe the identification of differentially expressed microRNAs in human male breast cancer using fluorescence labeled bead methodology. Several microRNAs are shared between female and male breast cancer, but our data also suggest that clear differences exist between breast cancer arising in males and females. Results were confirmed in the training set and an independent test set using real-time PCR methodology.

## Methods

### Patient Samples

21 specimens from 20 male patients with the clinically and histopathologically confirmed diagnosis of breast carcinoma in the period from 1995 to 2008 were selected from the archive of the Institute of Pathology, Medizinische Hochschule Hannover, Germany and analyzed anonymously following the guidelines of the local Ethics committee ("Ethik-Kommission der Medizinischen Hochschule Hannover", head: Prof. Dr. Tröger). For the comprehensive profiling of 319 microRNAs (trainings set, n = 9) only tumors with a size greater than 0.5 cm and a total RNA yield of at least 10 μg were selected. If tumor cell content was below 75%, tumor cells were manually microdissected from unstained histological sections using an H&E stained serial section as guidance. For a detailed description of all carcinoma cases see Table 1.

As a reference for normal expression level the RNA isolated from 15 male age-matched gynecomastia specimens was pooled into four control samples ("mix 1 - 4", made up of three or four individual RNA preparations, respectively) because the average RNA yield was quite low due to the limited number of epithelial cells present.

### Immunohistochemistry

Multi tissue arrays were constructed as described in order to ensure homogenous staining quality for all specimens [28]. Two tissue cores per specimen were analyzed and scored in a blinded fashion. Mouse monoclonal anti-ER (clone: 6F11), anti-PgR (clone: 16), and anti-HER2 (clone: CB11) antibodies were purchased from Ventana (Illkirch, France) and mouse monoclonal anti-CK5/6 (clone: D5/16 B4), anti-p63 (clone: 4A4), anti-MIB-1/Ki 67 (clone: MIB-1), and anti-androgen receptor (anti-AR; clone: AR441) antibodies were purchased from Dako (Glostrup, Denmark). The antibodies used for immunohistochemistry formalin-fixed, paraffin-embedded sections (3 μm) were deparaffinized in xylene and dehydrated through graded alcohols. Slides were rinsed thoroughly with Protein Blocking Agent (UltraTech HRP Streptavidin-Biotin Detection System, Beckman Coulter, Fullerton, CA, USA). Endogenous peroxidase was blocked in 3% hydrogen peroxide (H_2_O_2_) in methanol for 15 min. Antigen retrieval for ER, PR, HER2, CK5/6, p63, MIB-1, and AR was done by autoclaving at 121°C for 10 min in 0.1 mol/L citrate buffer (pH 6). The sections were incubated with one of the following antibodies: mouse monoclonal anti-ER antibody (predilution), anti-PgR antibody (predilution), anti-HER2 antibody (predilution), anti-CK5/6 antibody (1:100 dilution), anti-p63 antibody (1:50 dilution), anti-MIB-1 antibody (1:50 dilution), and anti-AR antibody (1:50 dilution) for 60 min at room temperature. Then, the secondary biotinylated goat polyvalent antibody (Beckman Coulter) was applied for 10 min at room temperature. The sections were incubated with peroxidase-conjugated streptavidin for 10 min and the reaction products were visualized using 3,3'-diaminobenzidine tetrahydrochloride (DAB) and H_2_O_2_. Counterstaining was performed using hematoxylin. The slides were rinsed with PBS after each stage of the procedure. As a negative control, all staining was also performed without the first antibody. When >5% of the tumor cells were stained with the antibody for p63 and CK 5/14, it was categorized as positive for antigen expression. HER2 staining was scored following the HER2 testing guidelines for breast cancer. ER, PR and AR expression was evaluated by the Allred score.

### RNA isolation

Isolation of total RNA from FFPE specimens was performed as described previously [29]. Following the recommendations from Luminex Inc. no microRNA enrichment was performed.

### microRNA measurement using fluorescence labeled beads

Quantification of microRNAs hybridized to fluorescence labeled beads was performed with a BioPlex 200™ from Biorad (Bio-Rad Laboratories GmbH, München, Germany) using the software Luminex IS™, version 2.3 from Luminex (Austin, Texas, USA). All beads coated with LNA probes complementary to mature microRNAs were purchased from Luminex Inc. (Austin, Texas, USA).

Prior to hybridization total RNA is labeled with biotin which is later bound to a streptavidin-phycoerythrin conjugate. For this purpose the FlexmiR™ MicroRNA Labeling Kit from Luminex Inc. was used. For the comprehensive profiling of 319 microRNAs (FlexmiR™ human microRNA pool, version 8) 2.5 μg total RNA were labeled following the protocol provided by the manufacturer. The 319 different microRNA beads are divided into 5 pools because there are not enough different fluorescence labels available to distinguish more than 70 - 80 beads in a single tube. For every pool 0.5 μg of total RNA are required. Subsequent washing, hybridization and analysis of samples were performed exactly as described in the protocols provided by Luminex Inc.

The system was calibrated using the xMAP™ calibration control reagents from Luminex Inc. following the recommendations of the manufacturer. Every run contained a negative control for background subtraction (water instead of RNA) and a positive control (total human brain RNA from Ambion, provided by Luminex Inc.).

### microRNA measurement using real time-RT-PCR technology

For the measurement of the expression levels of selected microRNAs (mir-21, -145, -497, -519d) the real time-RT-PCR-based detection methodology from ABI (Darmstadt, Germany) was used. Relative expression levels were calculated using the constitutively expressed small RNAs RNU24, U54, and Z-30 as endogenous controls. All primers and probes were purchased from ABI. cDNA synthesis and real-time PCR were performed following the protocols supplied by the manufacturer.

### mRNA measurement using real time-RT-PCR technology

For the measurement of the expression level of selected mir-21 target genes total RNA was transcribed to cDNA as described in ref. [30]. For this purpose an aliquot from the RNA preparation used for microRNA profiling was used. Subsequent real-time RT-PCR using SybrGreen as detection reagent was performed as described in ref. [31] with the same reference genes (βGUS and TBP). The primer sequences for the miR-21 target genes were taken from ref. [9].

### Statistical analysis

The raw intensity values were processed according to Blenkiron et al. [26]. Values smaller than 1 were equalized to 1. All values were log transformed to a basis of 2. The background values derived from the negative control were subtracted from all sample signals. A pool to pool probe effect normalization was performed as described by Blenkiron et al. [26]. Samples and miRNAs were clustered unsupervised hierarchically with the Eisen cluster software (EisenLab Cluster version 2.11) using average linkage and uncentered correlation. Visualization was performed with TreeView (version 1.60).

To compare the two groups (normal vs. carcinoma) Welch based t-tests were carried out using R statistical platform (version 2.80) and visualized using TIGR software (MeV version 4.3, http://www.tm4.org).

The correlation between microRNA expression and relative PCR C_T_-values was determined by Pearson product moment correlation.

## Abbreviations

AR: androgen receptor; C: control; DIC: ductal invasive carcinoma; ER: estrogen receptor; FFPE: formalin-fixed paraffin-embedded; rec: local recurrence; MBC: male breast cancer; Met: Metastasis; MIB: Ki-67 labeling index determined using antibody MIB-1; miR: microRNA; pap: papillary; PR: progesterone receptor.

## Competing interests

The authors declare that they have no competing interests.

## Authors' contributions

UL, HK, and FL conceived the study, BH, ES, and UL performed all measurements and evaluated the raw data, TS and BO performed the statistical analyses, UH, HK and FL selected and evaluated all cases, UL and FL wrote the manuscript with support from CA, HK, UH, TS, and BO. All authors approved the final manuscript.

## Pre-publication history

The pre-publication history for this paper can be accessed here:

http://www.biomedcentral.com/1471-2407/10/109/prepub
